# “World’s Fair,” from Dental News Letter

**Published:** 1851-10

**Authors:** J. R. McCurdy


From the Dental News Letter.

LONDON, June 5, 1851.

*Gentlemen:*—As promised in my last, I can now give you some slight description of the articles of dentistry on exhibition at the "World's Fair;" but I fear you may think it very meagre. If so, I can only say the display is meagre—far, far short of my anticipations. Dentistry in Europe affords but a limited subject for a long letter, for the reason that the mass of the work is after the same style, although with different degrees of finish, and in describing one collection all are described.

In all my researches through the exhibition, I found the following:

One case only from Switzerland, which consisted chiefly of bone work, or teeth carved from the hippopotamus, porcelain teeth on bone bases, and one or two cases of French teeth, on plates which were very narrow, and the soldering very rough. The clasps, from their arrangement, were well calculated to injure or destroy the teeth which they embraced. There was one case of human teeth, mounted on gold plate, which was neatly done, and an apparatus for regulating the teeth, which was very complicated and cumbersome, and which was to be attached to the teeth with strings. I may say of the bone work that it was neatly and ingeniously carved.

I may say here that I formed the impression that spiral springs are used to a much greater extent here than with us.

From France I could find but one specimen case, which, for size and the quantity of work it contained, was quite sufficient to represent the whole "Republic." At a venture, I would say there were at least fifty different specimens in the case, among which were some nine full cases, in active operation, chewing away lustily; also an entirely new plan of exhibiting, to the best or worst advantage, the want of teeth, after this fashion: as the jaw opens, two or three teeth in the upper jaw slowly move out of sight, leaving an ugly space, particularly remarkable when the jaws come together, then when the jaws open again, these two or three teeth come back to their place, and show the beauty of a perfect set of teeth. Another case is made to revolve slowly, by pivots in the sides, thus showing the shape and workmanship all around, also a great number of small pieces of bone work, or French teeth, on very narrow gold plates, and the clasps made by continuing a small strip of the plate to bend around the adjoining teeth, all beautifully finished and showing much ingenuity, but very unsubstantial and temporary. This collection was a fair exhibit of the majority of French workmanship, only of better finish; and just such cases, only smaller, may be seen at the doors of most of the dentists in Paris, besides numerous flaming placards on the walls, and abundant advertisements in the newspapers, setting forth the superior abilities of Monsieur so and so, surgeon dentist.

From Prussia, one case containing specimens of artificial teeth and samples of the material, which were very similar in style to those made by the French, but hardly so well formed. As some of your readers may not have seen the French teeth, I will endeavor to give a description of them. They present the appearance, and are about the thickness of the American teeth, but instead of round pins in the back for soldering, they have three narrow pieces of platina plate, two on one side, and one on the other of a groove which runs longitudinally along the back of the tooth to nearly the cutting edge. In mounting them, a pin suiting the size of the groove, is soldered to the plate upright, and the slips of platina plate imbedded in the tooth are bent over the wire and soft soldered. This makes a clumsy piece of work, and must be uncomfortable to the wearer, because they present such a rough and uneven surface to the tongue; besides, they are not, as may well be imagined, very strong, mounted in this manner; and again, the teeth are very opaque and unnatural in appearance.

Passing over a case or two of no possible interest, I come to the English collection, which is more full than that of any other country.

There are some fifteen cases containing artificial teeth mounted, most of which are, however, bone work. I find that a great proportion of artificial teeth in this country is of this hippopotamus bone, all of which are beautifully carved and very accurately fitted, showing great ingenuity in their adaptation and skill, and rapidity in carving. That "practice makes perfect," is abundantly proven in this branch, and I give them credit for beautiful bone work, as well as highly finished plate work. It is either a misfortune or good fortune, that all this kind of work has to be done over once a year, at most, which would not suit us Americans, as we could not spare the time, and would not like to spend the means, to have a new operation yearly. One argument used here in favor of bone work is, that there is no grating sensation experienced by the wearer, as is the case, to some slight extent, with porcelain teeth; but I think this objection to porcelain teeth would soon cease, if persons would but wear them a short time, and who would not prefer some slight temporary annoyance with porcelain teeth, to the extreme unpleasantness, if not filthiness, of bone teeth? I would, at least, and I speak knowingly, and I think all who wear bone teeth would, if they but knew the difference in point of cleanliness and permanency. Another plan here is, to mount both porcelain and natural teeth on bone bases and lastly, porcelian and natural teeth on gold and silver plate; however, the latter material is not often used. All the porcelain teeth manufactured in England are, as many of your readers know, made as thick, if not thicker than the natural organs, with holes through, bushed with gold or platina, a few, however, without any metal lining. In mounting them, the pins on which the teeth set, are adjusted and soldered to the plate in the proper position; previously, however, the teeth are ground to fit the plate accurately, and in this, as well as in bone work, the fit is complete, no space being left for the accumulation of food or other substance. After the pins are all arranged and soldered to the plate, they are coated with melted sulphur, and the teeth are slipped on and pressed to their position, when the sulphur hardens, thus holding the tooth tolerably firm. Another method is, to wrap the pin, previously made rough, with floss silk, and force the tooth on, the tube in the tooth having been roughened also.

I had always looked upon this method of making and setting teeth, after contrasting it with the American mode, as temporary, and I confess my opinion has not changed after examining the variety on exhibition in London.

All the plate work is beautifully gotten up, very highly polished, and neat in all particulars.

I noticed artificial porcelain teeth, deposited by two manufacturers in London, all finely finished and of natural shapes, but opaque, without the translucency of the best American teeth, and too thick; for, when worn in the mouth, they would I think, fill it uncomfortably full, and confine the tongue to too small limits.

There were several appliances to dentistry, in the shape of a "universal drill for removing decay in the teeth," at an angle of forty-five degrees, which was worked by a crank in the handle. An "electric galvanic apparatus for dental purposes," which was more complicated than necessary. "A compress for alveolar hæmorrhage," which was arranged to pass over and around the head, well calculated to remind one of a straight jacket or a dog's muzzle in dog days, but not half as important or necessary as either. Also, "a series of mechanical adaptations for regulating and preventing the irregularities of the permanent teeth." There was a collection of gold arrangements for capping, banding, etc., rather clumsy, to my notion, and not to be compared, in effectiveness and convenience, to the application of the spiral spring to the same purpose.

Also, "rotary scissors and knife, for dividing nerves," suggestive of the ligamentum dentis. Also, a mechanical leech, which struck me as being quite suitable for dental purposes, doing away with the repulsive crawling live leech.

I was particularly pleased with a series of experiments which were exhibited, showing the unfitness of bone work and silver plates. This was done by subjecting them to the action of dilute acid, which was dissolving them at a slow rate, then, with a written card explaining the whole matter to the spectator, telling him that the acid in the saliva would likewise decompose the bone and oxydize the silver, though not so rapidly. Many visitors were informing themselves on the subject, and I cannot but think that these public experiments will materially assist in doing away with that temporary description of dentistry.

I noticed some few cases of teeth mounted upon tortoise shell and gutta percha bases, but the first substance, I was informed, would not retain the shape which was given it by heat, but had a tendency and would gradually return to its original shape. The gutta percha was too soft and yielding, especially so when at the temperature of the mouth.

We now come to the collection from the United States, which I sum up briefly. Two cases of block teeth, mounted. One case of blocks, not mounted. Three cases of gold foil, one of which is from Jones, White & McCurdy. Five cases of mechanical dentistry. One case dental instruments. One case tooth wash and dentrifice. Three cases of artificial teeth, one of which is from Jones, White & McCurdy. And last, though not least, two cases of plugged teeth, one of which is marked simply "Philadelphia," and is, unquestionably, among the best and prettiest fillings I ever saw. They reminded me forcibly of a certain gentleman's workmanship, but whether his or not I would not like to say. However, Philadelphia has the honor of it, which is no small praise. The other case is, to all appearance, very creditable, and they both reflect much honor upon American dentistry.

Of the American teeth here, it, perhaps, does not become me to say much; but this I may say, and I think it is evident to any unprejudiced mind, that they, or some of them, are much more translucent and vital in appearance, more beautifully tinted, and more natural in shape and shade, than any others from any quarter. And when mounted, as it is done in our country, and shown here by several beautiful specimens, (all of which are from Philadelphia and New York,) they can be worn with more ease and comfort, and be more serviceable and permanent than any other style of teeth mounted in any other manner. I may be prejudiced, but I think a fair comparison, by competent judges, will prove the correctness of the above.

The amount of dentistry performed in England is quite limited, in comparison with our own country; for these reasons, probably, that their teeth generally are more durable. Again, it is the upper classes only who can afford it—while in France, their teeth are, to all appearance, quite as frail, and decay quite as soon, as with us; yet there, also, but few can afford to pay for it. In Germany their teeth look as if they would never know decay, and, consequently, the dentist gets but a poor support. I would not neglect to acknowledge the courtesy extended to me by many gentlemen in the profession, among whom I may mention Dr. James Robinson, of London, Dr. Mein, of Edinburgh, Dr. Brophy, of Dublin, Dr. Helsby, of Manchester, and Dr. Evans, of Paris. Many others I might mention, but they will all receive my hearty thanks, and I can only say, I hope I may have the opportunity of reciprocating, in some way. With Dr. Evans, who is an old acquaintance, I felt quite at home. And I will just say here, that America bears off the palm in dentistry; for Dr. Evans numbers among his patrons the Kings of Bavaria, Prussia and Greece, and the President of the French Republic, beside numbers of the nobility, among whom are several prominent persons at the Court of the Emperor of Russia, and all obtained through several difficult but successful operations performed for persons high in office and influence at some of the above-named courts. I wish him success, with all my heart, as he well deserves it.

To conclude this very long and desultory letter, I would say, that great quantities of amalgam are used in France and Germany, and much in England, and oftentimes the filling is put in without removing the decay. Low grades of gold plate are used, also some silver, and, occasionally, palladium.

The prices, so far as I could get at them, range about as with us, some getting high rates, aud some working very cheap for very cheap work. Occasionally, however, as with Drs. Brewster and Evans of Paris, a large sum is received for an important operation, when performed for an important personage.

In haste, yours, truly, J. R. McCurdy.

